# The potential of swimming pool rinsing water for irrigation of green areas: a case study

**DOI:** 10.1007/s11356-023-26126-x

**Published:** 2023-03-04

**Authors:** Wojciech Poćwiardowski

**Affiliations:** Faculty of Chemical Technology and Engineering, Bydgoszcz University of Science and Technology, Seminaryjna 3, 85-326 Bydgoszcz, Poland

**Keywords:** Rinsing water, Irrigation, Circular economy, Recovery water

## Abstract

The aim of the research was to check the possibility of using the rinsing water after rinsing the recreational pool filters, cleaned with the rinse water recovery system, for irrigation of green areas. The system consists of the following stages: flocculation, pre-filtration, and ultrafiltration based on filter tubes. The degree of contamination of the rinse waters before and after the treatment process was assessed by means of physicochemical and microbiological tests, and then compared with the permissible parameter values for wastewater discharged into the ground or water. Thanks to the use of flocculation and appropriate ultrafiltration, the high level of total suspended solids and total organic carbon was reduced, which allows for safe discharge of purified water into the environment.

**Graphical Abstract:**

Circular economy, wash water, zero waste technologies, water footprint, water recycling

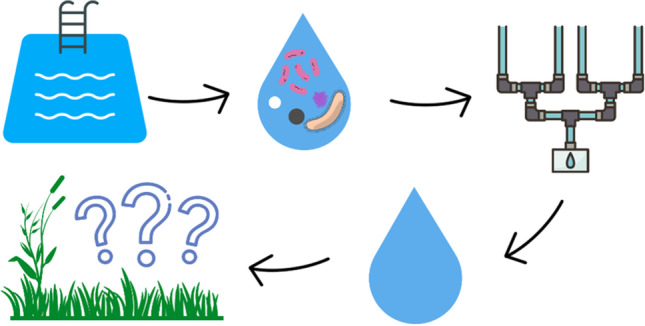

## Introduction

The existing water deficits, the need to save water and the growing prices of water and sewage have caused designers and owners of water treatment systems, such as swimming pool facilities or water treatment plants, to look for new solutions in the field of water and wastewater management. It is worth remembering that raw pool water (e.g.: brine) can not be directly implemented in the production of drinking water or industrial applications (Panagopoulos 2022; Panagopoulos and Giannika 2022a, b). The washings generated as a result of rinsing the filter beds of water treatment systems, depending on the water demand and the water treatment technology used, may constitute from 20% to even 70% of the total volume of wastewater. For the proper performance of the filter bed rinsing process, 4 to 6 m^3^ of water per m^2^ of bed is required (Sokołowski 1998; Deutsches Institut für Normung 2012; Chief Sanitary Inspectorate 2014; Alansari et al. 2018). The standard pool water treatment system, consisting of 2 filters with a diameter of 1800 mm, rinsed on average every 2 days, requires a monthly consumption of rinsing water with a volume of 245 to 365 m^3^.

Therefore, the aim of the research was to use an innovative system of rinsing water treatment from filter rinsing, based on flocculation, pre-filtration, and ultrafiltration with the use of filter tubes used for hemodialysis (Studziński et al. 2021), in a swimming pool facility and to check its effectiveness in removing microbiological and physicochemical contamination, and then analyzing the potential of the obtained water for other uses, including watering green areas.

## Materials and methods

The subject of the research were washings samples taken from the water treatment circuit feeding the recreational swimming pool in the indoor public swimming pool. The facility is characterized by a high load of 66.6 l/h and a high-water temperature in the basin of 30–32 °C. Sodium hypochlorite is used in the disinfection of the recreational pool.

The rinsing water were collected during the washing of sand and gravel beds of pressure filters, which are the main element of the swimming pool water treatment system and directed to a separate reservoir for further treatment with the use of a wash water recovery system. Purified water samples were taken with a sampling tap directly after the ultrafiltration unit and analyzed.

In order to determine the microbiological parameters of the tested water, reference methods of analysis were used (Ministry of Health 2015). Investigated microbiological parameters: *Escherichia coli*, *Pseudomonas aeruginosa*, coagulase-positive staphylococci, *Legionella* sp., *Faecal streptococci*, total number of microorganisms at 36 °C. The selected parameters comply with the guidelines of the current polish regulation of the Minister of Health of November 9, 2015, on the requirements to be met by water in swimming pools, Journal of Laws 2015, item 2016.

The analysis of the physical and chemical parameters of the tested water was carried out in accordance with the reference methodology for water analyzes in swimming pools. Tested physicochemical parameters: nitrates, ammonium ion, five-day biochemical oxygen demand (BOD at 20 °C), determined with the addition of a nitrification inhibitor, chemical oxygen demand (COD), determined by the bichromate method, free chlorine, total chlorine, total phosphorus, aluminum, general iron, turbidity, color, pH, temperature, total suspended solids, total organic carbon (TOC), total iron. The selected parameters comply with the guidelines of the current polish regulation of the Minister of Health of November 9, 2015 on the requirements to be met by water in swimming pools, Journal Of Laws 2015, item 2016 (Ministry of Health 2015). All tests were conducted in triplicate, and the results were averaged.

## Results

The laboratory tests carried out have shown that the rinse waters are waters with very variable parameters. They show a very high level of microbiological contamination (Table 1).

**Table 1:** Results of microbiological analyzes of washings before and after using the rinse water recovery system

Parameter	Unit	Raw water	Purified rinse water
Total number of microorganisms at 36 °C	[CFU/cm^3^]	1,62 × 10^4^	> 3,0 × 10^2^
*Escherichia coli*	[CFU/100 cm^3^]	0	0
*Legionella* sp.	[CFU/100 cm^3^]	0	0
*Pseudomonas aeruginosa*	[CFU/100 cm^3^]	2	0
Coagulase positive staphylococci	[CFU/100 cm^3^]	0	0
Faecal streptococci	[CFU/100 cm^3^]	0	0

The physicochemical parameters also indicated high contamination of the rinse water (Table 2). The water was characterized by high turbidity (> 200 NTU), color (336 Pt mg/dm^3^), total suspension, and total organic carbon (120 mg C / dm^3^).

**Table 2:** Results of physicochemical analyzes of washings before and after using the rinse water recovery system

Parameter	Unit	Raw water	Purified rinse water	Parametric value
Nitrates	[mg N-NO_3_/dm^3^]	4.43	4.40	30
Ammonium ion	[mg N-NH_4_/dm^3^]	2.826	0.182	10
Biochemical oxygen demand (5 days)	[mg O_2_/dm^3^]	9.1	2.4	15
Chemical oxygen demand	[mg O_2_/dm^3^]	62.3	1.04	125
Free chlorine	[mg Cl_2_/dm^3^]	0.05	0.02	0.2
Total chlorine	[mg Cl_2_/dm^3^]	0.08	0.02	0.4
Total phosphorus	[mg P_og_/dm^3^]	10.6	0.25	2
Aluminum	[mgAl^+3^/dm^3^]	< 0.020	< 0.020	3
General iron	[mg Fe/dm^3^]	0.317	< 0.010	10
Color	[mg Pt/dm^3^]	336	5	*
Turbidity	NTU	200.00	0.41	*
pH	-	7.1	7.3	6.5–9.0
Temperature	[°C]	26.4	25.0	35
Total suspended solids	[mg/dm^3^]	348	9.2	35
Total organic carbon	[mg C/dm^3^]	120.40	9.40	30

*No parametric values for these parameters in the regulation

**Fig. 1:**
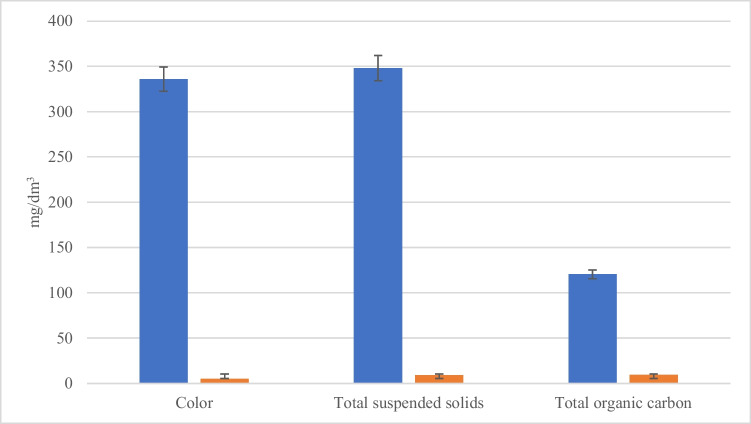
Changes in selected physicochemical parameters

Conducting treatment of the rinse water resulted in a notable decrease in turbidity values from 200 to 0.41 NTU, total suspended solids from 348 mg/dm^3^(standard: 35 mg/dm^3^) to 9.2 mg/dm3. The critical value of total organic carbon also decreased from 120.4 to 9.4 mg C/ dm^3^. There was also a significant improvement in the color of the water (Fig. 1).

## Discussion

The results of the research indicate a significant reduction of microorganisms after the application of the wash water treatment system (Table 1). On the other hand, the physicochemical parameters of water collected after ultrafiltration were also significantly reduced, especially problematic indicators such as TOC, total suspended solids, turbidity, total phosphorus, and COD (Table 2).

In accordance with the Polish Regulation of the Minister of Maritime Economy and Inland Navigation of July 12, 2019 “On substances particularly harmful to the aquatic environment and the conditions to be met when discharging into waters or soil, as well as when discharging rainwater or meltwater into waters or to water facilities” (Ministry of Maritime Economy and Inland Navigation 2019), it is not possible to use rinsing water directly for economic and agricultural purposes, due to the exceeding of certain parameters. The analysis and comparison of the washings test results showed that their direct discharge into watercourses or soil was impossible, mainly due to too large amounts of total suspended solids and total organic carbon (Table 2). Further analysis of the washings after the ultrafiltration process allowed to define the concept of their management through their discharge into water courses or their use for irrigation of green areas. The condition for such management would be the use of a flocculation tank to support the sedimentation of suspensions, and then discharge of concentrated sewage to the sanitary sewage system (Wiercik and Domańska 2011; Wyczarska-Kokot and Lempart 2019; Łaskawiec et al. 2019).

The presence of physicochemical and microbiological factors threatening human health and life in the recovered rinse water was not found. Additional reduction of the presence of *Pseudomonas aeruginosa* can be carried out using the method of ozonation of water.

Recovered water meets biological and physicochemical standards and can be used to irrigate crops. However, more research is needed on the reuse of washwater in agriculture and horticulture. This short article can be a prelude to further research on the implementation of zero-waste policies and a closed-loop economy.

## Conclusion

The rinse waters obtained from the tested object were characterized by very high turbidity, total suspended solids, as well as significant microbiological contamination. For this reason, it is not possible to discharge raw washings from the filters installed in the water treatment system of the tested swimming pool facility directly into the river or their infiltration into the soil.

The rinse water purification process carried out with the use of a water recovery system, and above all the flocculation process and the use of an innovative version of ultrafiltration, made it possible to obtain a largely purified washings, allowing them to be safely discharged into the environment and used for irrigation of green areas, as well as for industrial purposes.

Previous own studies also indicated the high efficiency of the implemented water treatment system. Recovered water has been used for circular supply in several swimming pool complexes in Poland. This demonstrates that reclaimed washwater can find applications in both industry and agriculture (Studziński et al. 2021).
